# Barriers and facilitators of successful weight loss during participation in behavioural weight management programmes: a protocol for a systematic review

**DOI:** 10.1186/s13643-020-01427-1

**Published:** 2020-07-30

**Authors:** Meigan Thomson, Anne Martin, Jennifer Logue, Valerie Wells, Sharon A. Simpson

**Affiliations:** 1 MRC/CSO Social & Public Health Sciences Unit, University of Glasgow, Glasgow, UK; 2 Lancaster Medical School, University of Lancaster, Lancaster, UK

**Keywords:** Weight loss, Behaviour change, Behavioural intervention, Barriers, Facilitators, Systematic review

## Abstract

**Background:**

Behavioural weight management programmes are effective in assisting people with overweight or obesity to lose excess body weight. Yet, many still struggle to attain their weight loss goals in such programmes. Little is understood about the factors which impact success in these programmes. Synthesising this data will allow for theory to be developed on how to improve success in such programmes. The main aim of this review will be to extract and synthesise the barriers and facilitators of successful weight loss during participation of behavioural weight loss programmes in adults living with overweight and obesity.

**Methods:**

A systematic search of MEDLINE, Embase, PsycINFO, The Cochrane Library and CINAHL will be performed from inception onwards. Studies will also be sought by contacting experts in the field, reference and website searching. Studies will be eligible if the participants are adults living with obesity (population) undertaking or recently completed behavioural weight loss programmes (intervention) with the primary focus of weight loss (outcome). The primary outcomes will be amount of weight lost and information on barriers and/or facilitators to success. The secondary outcomes will be reasons or factors related to attrition and adherence and behaviour change techniques used in programmes. Two reviewers will screen citations and full-text data. Reviewer 1 will screen all, and reviewer 2 will screen a random 50% of articles. Data extraction will be completed by reviewer 1, and 10% will be checked by the research team. Potential conflicts will be resolved through discussion. Data will be synthesised and described narratively to show the characteristics of each study, levels of success and barriers and facilitators during programme participation. A thematic approach will be taken, and themes will be coded against the levels of the socioecological model. Quantitative data will be extracted and categorised according to these themes and presented alongside the qualitative data.

**Discussion:**

Our findings can be used to inform how weight loss programmes can be improved to facilitate success in those at risk of failure. Results will be published in a peer-reviewed journal.

**Systematic review registration:**

(PROSPERO CRD42019148158 )

## Background

The prevalence of obesity has grown exponentially over the last few decades. Since the mid-1970s, the prevalence of obesity has increased from 3.2% in males and 6.4% in females to 10.8% in males and 14.9% in females in 2014 [1]. More recent data from the World Health Organization has shown that obesity prevalence has continued to remain relatively high with 11% of males and 15% of females living with obesity in 2016 [2]. It is anticipated by 2025, obesity rates could be as high as 18% in males and 21% in females [1].

Obesity is defined as excess body weight in the form of adipose tissue which presents a risk to the person’s health [3]. Obesity is associated with a reduction of healthy years across the lifespan and mortality due to health issues related to their obesity [4]. People living with obesity report experiencing a lower quality of life on physical and mental health subscales [5]. Obesity is associated with an increased risk of developing type 2 diabetes, cancer, cardiovascular disease, depression and dementia [6–9]. The risk of developing such conditions and prognosis after diagnosis (e.g. cardiovascular disease, depression) can be alleviated through weight loss. Evidence shows a reduction in weight of 5% can improve outcomes [10, 11].

Behavioural weight management programmes have shown to be efficacious in supporting people to achieve a > 5% weight loss [12, 13]. These programmes are interventions which aim to support participants to change their eating and physical activity behaviours. In the context of weight loss, such programmes support people to change their dietary and physical activity behaviours through education and behavioural change techniques [14, 15]. Behaviour change techniques used vary from programme to programme but typically involve elements of self-monitoring (e.g. keeping a diary of food consumed or triggers for unhealthy eating), planning (e.g. creating meal plans or meal preparation) or problem solving (helping individuals identify possible problem situations, moods and influencers and create a plan to deal/cope with the problem) [14, 16, 17].

Participants enrolled in such programmes on average lose twice as much weight as those in standard care and are more likely to reach the 5% weight loss goal [13]. Yet, randomised controlled trials commissioned by public health services have shown that up to 50% of those attending these programmes do not achieve 5% weight loss [13, 18]. Researchers have conducted systematic reviews of the literature to identify factors impacting and predicting adherence, drop-out and success in specific groups in these programmes [17, 19–21]. However, to our knowledge, none of the existing systematic reviews synthesise the qualitative and quantitative data related to barriers and facilitators of weight loss during participation in these programmes. The rationale for this review is despite our current knowledge on predictors of success, dropout or adherence, many still fail to reach their goals in such programmes. Uncovering the barriers and facilitators participants face during their participation will add to the current knowledge base and provide further guidance on how we can improve success during the programme. Such information could inform theory and the design of such interventions to improve success and retention rates. The Foresight report showed the causes of obesity to be a complex interplay of biological, social, psychological, environmental, policy and community level factors [22]. We, therefore, theorise a similar interplay of factors in the context of weight loss in behavioural weight management programmes. For the scope of this review, we anticipate being able to extract data on individual, social/community, environmental, cultural and policy and programme-specific level factors.

The primary objective of this systematic review is to synthesise the existing evidence on barriers and facilitators (individual, social/community, environmental, cultural and policy and programme-specific level factors) to success while participating in behavioural weight management programmes. The secondary objectives are (1) to explore evidence related to factors influencing adherence and attrition during participation and (2) to extract information on which behaviour change techniques are used in programmes with higher success rates and compare this to programmes with lower success rates.

The aim of this systematic review is to develop a fuller understanding of what contextual, social, individual and programme-specific factors impact on weight loss success and failure and allow theory to be developed on how programmes can be improved to support those who fail to achieve a 5% weight loss.

## Methods

The present protocol has been registered within the PROSPERO database (registration number CRD42019148158) and is being reported in accordance with the reporting guidance provided in the Preferred Reporting Items for Systematic Reviews and Meta-Analyses Protocols (PRISMA-P) statement [23] (see checklist in Additional file 1).

### Eligibility criteria

Studies will be selected according to the following criteria: population, interventions, comparators, outcome(s) of interest and study design (PICOS). The inclusion and exclusion criteria can be found in Table 1. There will be no restrictions on dates of publication. Due to the resources attributed to the project, only articles in English will be included, and searches will be restricted to 5 databases.

**Table 1:** Inclusion and exclusion criteria.

PICOS domain	Inclusion	Exclusion
Population	18+ years Living with overweight or obesity, defined as one of the following weight measurements: • BMI > 25 • Waist circumference (+ 88 female, + 102 males)	Pregnant women Post-surgery patients Inpatients Studies where the entire population have one of the following: • Mental health condition • Learning disability • Presence of a physical health/disease condition which could impact the content of the intervention
Intervention	Behavioural weight management programmes Use of behaviour change techniques Focus/goal is weight loss	Studies and interventions whose primary goal is not weight loss or have been altered to include more than a weight loss element, or are aimed at groups where the intervention would need to be adapted for use Studies only testing diet or meal replacements without a behaviour change element Studies targeting multiple health problems at once (i.e. obesity and alcohol use) Studies focusing on weight gain prevention
Comparator/outcomes	Weight outcomes (baseline and end of participation weights or %/kg lost) Behaviour change techniques used (explicit or described) One of the following: • Information on barriers/facilitators • Participant feedback on the intervention	
Study	Intervention studies Qualitative interviews Qualitative focus groups Process evaluation studies Set in Western high- and middle-income countries	Case studies Systematic reviews

#### Population

Studies involving adults (18 + years) will be included. Weight management programmes which have been tailored for a specific population based on their specific needs will be excluded. If studies only have the following populations, they will be excluded: pregnant women, post-surgery patients, inpatients, mental health disorders, learning disabilities or the presence of a physical health/disease condition (e.g. cancer, diabetes). It is anticipated that these programmes will have been tailored beyond the usual role of a weight loss programme and thus would be less relevant to identifying factors impacting a typical behavioural weight loss programme.

#### Intervention

The review will include studies involving behavioural weight management programmes (utilising behaviour change techniques for weight loss) with the primary goal of weight loss. Interventions will be excluded if their primary objective is not weight loss, or they have been altered to include more than a weight loss element or are aimed at groups with specific health or psychological needs (e.g. adapted for disease education, comprehension or specific physical needs). For example, weight loss interventions for individuals with diabetes will be excluded as such programmes involve education in the domain of diabetes and have diabetes-specific aims and outcomes which are beyond that of weight loss. Weight loss programmes included can be of any duration and will be compared by duration/number of sessions in the presentation of results.

#### Control/comparator

Weight loss outcomes of participants will need to be reported to allow for comparison between studies and any factors associated with successful weight loss (5% reduction from baseline) to be identified.

#### Outcomes

The primary outcomes will be barriers and facilitators of success in behavioural weight loss programmes. These will be modifiable factors which the participants experience during participation in the programme. Barriers may include family and time commitments, lack of social support, aspects of the programme or the individual’s motivation/enjoyment of the programme. Facilitators may include feeling part of the programme, high levels of social support, seeing weight loss results early in the programme or the individual being more adaptable. Studies which only report demographic outcomes and their relation to success will not be included as the focus of the review are factors impacting success in programmes for the general population (accessible to any age, sex, race or income). This data will be in the form of patient or facilitator feedback (collected either qualitatively through interviews or focus groups or quantitatively via surveys or measurements of factors like motivation), study results where success rates are linked to certain variables (e.g. motivation for weight loss) and any available process evaluation data (e.g. programme-specific evaluations and their relationship to success) collected during the study or at follow-up of up to 3 months. A 3 months cutoff has been defined for follow-up to try and ensure factors identified are related to weight loss and the intervention itself rather than issues related to longer-term maintenance.

The secondary outcomes will only explore data collected during programme participation. The review will compare characteristics of programmes with high and low success rates and extract data on behaviour change techniques used and adherence and attrition rates. Any data (quantitative or qualitative) on reasons for adherence or attrition will be extracted and compared. This can either be in the form of quantitative data such as participant characteristics (e.g. sex, motivation, age, socioeconomic status) or survey feedback or qualitative feedback given by participants in interview or focus groups from trials.

To be eligible for inclusion, studies will also need to have reported a clear measurement of either weight at baseline and end of participation or the amount of weight lost over the course of the programme and include data on barriers or facilitators faced during the programme. Qualitative studies included in the review will be from weight management programme trials, where we expect weight loss data to be reported either within the article or potentially in additional reports of the same study. Studies only reporting follow-up data after a programme has been completed will be included, but we will impose a limit of up to 3 months from the end of the programme, as we believe data beyond this time point is less likely to be linked to factors impacting participants during participation in a programme.

#### Study designs

We will include experimental, quasi-experimental, qualitative and mixed-methods studies. Only studies which are set in Western high- and middle-income countries will be included. This might allow comparison between barriers/facilitators due to higher levels of homogeneity between contexts. These countries will be identified using guidance from the Organisation for Economic Co-operation and Development (OECD): http://www.oecd.org/dac/financing-sustainable-development/development-finance-standards/daclist.htm. Case studies, systematic reviews, and protocols will also be excluded. Case studies of participants will be excluded due to issues with generalising beyond the study. Systematic reviews will be excluded, but the references will be reviewed for further eligible studies.

### Information sources and search strategy

We will search the following databases (from inception onwards): MEDLINE, Embase, Cochrane Library, PsycINFO and CINAHL. The initial search strategy was developed by MT supported by VW and AM to reflect the eligibility criteria. We used the population, intervention, comparison, outcome and study (PICOS) format to develop our search terms [24**]**. The MEDLINE search strategy can be found in Additional File 2. This version of the search strategy will be tested on MEDLINE to assess the number of results received and assess if the search is thorough enough. Two reviewers (MT and AM) will take a random selection of 50 results each and will test these against the developed eligibility criteria and review the results for additional relevant search terms and update the search strategy as needed. Additionally, other systematic reviews and keywords in papers within the domains of obesity and weight management were used to develop a thorough strategy.

All results will be downloaded and stored in Endnote (https://endnote.com/). Endnote will be used to remove duplicates, and the remaining will be uploaded to Covidence (https://www.covidence.org/home) for screening and data extraction.

Additional relevant studies will be sought through the following methods:

- Screening of reference lists:
  - ○ Systematic reviews found in the search will be assessed for their relevance to the research question using phase 1 of the Risk Of Bias In Systematic reviews tool (http://www.bristol.ac.uk/population-health-sciences/projects/robis/). Those considered relevant will have the citations for the studies included in their reviews downloaded into Endnote. This will be recorded in the PRISMA diagram. Any duplicates from our previous search results will be removed, and the remaining papers will be uploaded into Covidence for screening.
- Website searches:
  - ○ Public Health England (https://www.gov.uk/government/organisations/public-health-england)
  - ○ The Scottish Public Health Observatory (https://www.scotpho.org.uk/)
  - ○ Association for the Study of Obesity (https://www.aso.org.uk/)
  - ○ European Association of Obesity (https://easo.org/)
  - ○ Centre for Disease Control and Prevention (https://www.cdc.gov/)
  - ○ World Health Organization (https://www.who.int/)
- Contacting experts in the field of adult weight management.
  - Experts will be identified through the screening process and by those identified through the above websites. Corresponding authors for any protocol papers or conference abstracts for studies will be contacted. They will be contacted via email and asked if there are any full texts of their work or preliminary data. Any published full-texts works will be included in the review.
  - A Twitter call will also be made for literature. We will set up a form that can be shared via Twitter, and respondents can fill in details/upload documents to be considered for the review. This call will be shared via the networks of the researchers in the study. We will use the following hashtags: #obesity #research #weightloss #weightintervention #systematicreview. A log of retweets and number of responses will be kept. Data received through this method will be explicitly recorded in the PRISMA diagram.

### Screening and data extraction

All screening will be conducted in Covidence. Preliminary screening of studies will be based on information contained in the titles and abstracts. All screening will be conducted by MT with a random selection of 50% being screened independently by a second reviewer. Any disagreements between MT and the second reviewer will be discussed between the researchers, and if agreement cannot be made, this will be discussed with the wider research team (AM, JL, and SS). Full paper screening will be conducted by the same researchers (all by MT and a minimum of 50% by the second researcher) with disagreements resolved within the wider research team (AM, JL, SS).

A data extraction form will be developed and piloted and will be used to extract data from the included studies on weight outcomes, barriers and facilitators experienced, behaviour change techniques, levels of adherence and attrition used in each programme. Data on the format, duration and location of each programme will be extracted and compared on success rates, adherence and attrition. Information on described or explicitly stated behaviour change techniques will be extracted from each paper and categorised using Michie et al.’s taxonomy of behaviour change [25]. Table 2 shows the data which will be extracted from each eligible study. Data extraction will be conducted by MT, and a second reviewer will check the extracted data. To recognise the complexity of weight loss, we anticipate the data extracted will inform the role of individual, social/community, environmental, cultural and policy and programme-specific level factors.

**Table 2:** Data to be extracted from studies

Domain	Data
Publication details	Author Year Funder
Participant characteristics	Age Sex Socio-economic status/income/occupation
Programme/intervention characteristics	Number of sessions Time period Length of each session Format of sessions (group, activities, educational, online) Behaviour change techniques used
Outcomes	Weight outcomes (baseline & end of programme, or amount lost) How weight is determined (measured or disclosed) How outcomes were assessed Levels of attrition Levels of adherence
Comparison of successful and unsuccessful	Identified barriers or facilitators (either through process evaluation data or participant feedback) Differences between successful/unsuccessful (i.e. attendance rates, social support, compliance, baseline characteristics)

### Risk of bias assessment

Risk of bias assessments will be conducted by MT and another member of the research team. This will be split between the research team (SS, AM, JL) who will check these assessments for all included studies. The following appraisal tools from the Critical Appraisal Skills Programme (CASP, 2010) (https://casp-uk.net/casp-tools-checklists/) will be used to assess the included studies:

- CASP qualitative checklist
- CASP randomised controlled trial checklist
- CASP cohort study checklist

### Data synthesis

The data from the eligible studies will be synthesised narratively. The synthesis will combine both qualitative and quantitative data. We anticipate most of the extracted data will be qualitative in nature, and where quantitative data is available there will be variability in what factors are assessed leaving limited scope for meta-analysis. It is expected that terminology and measurement of barriers and facilitators will vary between studies, and to synthesise this evidence, a narrative approach will be needed.

#### Study descriptions

We will describe each study by extracting and presenting the following information:

- Study population (age, sex, socio-economic status)
- Characteristics of the intervention (format, duration of sessions, content, behaviour change techniques used)
- Weight loss outcomes (comparison of baseline and end of intervention weights or amount of weight lost)
- Barriers and facilitators experienced
- Recommendations

Summary tables will be created to provide an overview of the structure and content of each included intervention. Behaviour change techniques will be identified in the text either where the authors have explicitly named the technique or given a relevant description of the technique being used and will be labelled according to Michie et al.’s taxonomy of behaviour change [25]. Where the behavioural change techniques used are not clear, we will contact authors for further information by email. Baseline weights and end of programme weights (or data on amount of weight lost) will be extracted from each study alongside adherence and attrition rates. The weight outcome data and adherence/attrition rates will be used to evaluate whether studies have high success rates or not, and a narrative comparison of studies with high/low success rates will be made. Study interventions will be compared on their structure, format and behaviour change techniques used. Finally, any quantitative and qualitative data on barriers and facilitators experienced during the programme will be extracted. This data can be in the format of qualitative feedback (i.e. interviews or focus groups with patients or facilitators), quantitative feedback (surveys), study results (e.g. if certain variables are attributed to success) or participant/contextual characteristics identified by authors as impacting success (e.g. having children, working).

#### Qualitative data

Any participant feedback, interview or focus group data relating to what impacted their experience in the weight loss programme will be extracted. The extracted qualitative data on factors impacting success will be analysed thematically. Data will be coded using a thematic synthesis approach [26]. The data extracted will be coded into factors associated with barriers and facilitators to weight loss in these interventions. Similar codes will then be grouped together to create descriptive themes which encompass several of the codes. Finally, analytical themes will be created to categorise the barriers and facilitators. These analytical themes will be split between the overarching themes of “barriers” or “facilitators” then the overarching themes within this will be coded against the levels of the socioecological model in line with our aims to explore the following: individual, social/community, environmental, cultural and policy and programme-specific level factors influencing success within weight loss programmes. Any other relevant overarching themes which emerge from the analyses will be included. Each overarching theme will have a set of sub-themes to explore the constructs within this which impact success.

#### Quantitative data

For all quantitative data, we will follow the most recent guidance on how to report the synthesis of data which cannot be combined a meta-analysis [27]. Any quantitative data reported in papers as being a barrier or facilitator to success or failure during programme participation will be extracted. We expect the collected quantitative data to vary between studies since we are not seeking a single measurable construct and will extract any information on barriers and/or facilitators. We will aim to merge this with the qualitative data we extract. To do this, quantitative data will be converted into a qualitative item to incorporate into the analysis of the results. Depending on the content of the papers, this will be operationalised in one of two ways.

1. If the authors of the paper state an interpretation of the variable and its relationship to success, this information will be extracted.
2. If such information is not stated, MT will write a short interpretation of the result. This will be reviewed by the supervisory team (AM, JL, SS)

Where it is not clear how the quantitative variable impacted success, this will not be transformed into a qualitative item and will be reported in the summary table and labelled as a factor which could not be categorised as a barrier, facilitator or both.

#### Integration of quantitative and qualitative data

All data will be imported into NVivo for analysis. These themes and their relationship to success will be discussed in the text and summarised in a table. The table created will contain a row for each study and a list of the identified barriers and facilitators and which theme they are coded to. The quality assessment scores will be included in a table to show the strength of the evidence for each study. The themes identified will be used to develop theory and a conceptual map to describe the factors in weight loss programmes that seem to impact on success. The quality assessment scores will be used to inform the details and weightings of different factors in the theory and conceptual map.

## Discussion

The procedures described in this protocol aim to provide a synthesis of the existing evidence on barriers and facilitators to weight loss in behavioural weight management programmes. We have presented a transparent and detailed explanation of how we aim to collect, extract and analyse this evidence. There are some limitations to our methodology. We will not be able to explore the impact of biological factors on weight loss success. However, as per previous evidence, we expect other factors to be more important or act as drivers of obesity [22, 28, 29]. We also anticipate that there will be limited scope for collecting evidence on community factors, as it is unlikely that most studies will explore these in detail. Identified factors may also act as both a barrier and/or a facilitator creating a problem in categorising influencing factors in this way, for example a programme being delivered in a group setting may act as a facilitator in the sense that it creates motivation and peer-support but also as a barrier if a person who is not losing weight disengages because of other people doing better than themselves. When we report the results, we will highlight this where appropriate. We expect due to the nature of the included studies, many of the influencing factors will be identified through the subjective experience of participants. This will give us potentially novel and useful insight into what factors participants themselves view as impacting their journey. This may not give full insight into the wider range of influences on their behaviour and experiences. Categorising the quantitative data in a qualitative way may be challenging. However, we hope by following our protocol this will be achievable. In cases where it is difficult to interpret how the data impacts success, we will report this in a table.

## Dissemination

The results of this review will be submitted for publication via open access route to ensure access to the wider public. The results will also be submitted as part of a PhD thesis and presented at conferences.

## Supplementary information

**Additional file 1.** PRISMA Checklist.

**Additional file 2.** MEDLINE Search Strategy.

## Data Availability

Data sharing is not applicable to this article as no datasets were generated or analysed during the current study.
