# Supplementary material for: Barriers and facilitators of successful weight loss during participation in behavioural weight management programmes: a protocol for a systematic review
Source: Syst Rev. 2020 Jul 30;9:168. doi: 10.1186/s13643-020-01427-1 (PMC7393897; doi:10.1186/s13643-020-01427-1)
Supplement: Supplementary file 2 — Additional file 2. MEDLINE Search Strategy. [file 13643_2020_1427_MOESM2_ESM.docx]

**Additional File 2:** MEDLINE Search Strategy

| **Row** | **Search Item** |
| --- | --- |
| 1 | Obesity/ or Obesity, Morbid, Abdominal/ |
| 2 | Exp weight gain/ |
| 3 | Overweight/ |
| 4 | (overweight or over weight or overeat* or over eat).ti,ab. |
| 5 | (weight adj1 gain*).ti.ab. |
| 6 | (weight adj1 loss*).ti,ab. |
| 7 | Obes*.ti,ab. |
| 8 | 1 or 2 or 3 or 4 or 5 or 6 or 7 |
| 9 | (modific* or therap* or intervention* or strateg* or program* or management or scheme* or group* or pathway*).ti,ab. |
| 10 | (Behavioural or behavioral or group or lifestyle or psych* or therap* or support or commercial or plan or project or non surgical or non-surgical or coaching or weight watchers or weightwatchers or WW or slimming world or Jenny Craig or counseling or counselling).ab,ti. |
| 11 | (weight adj1 los*).ti,ab. |
| 12 | (weight adj1 reduc*).ti,ab. |
| 13 | weight loss/ |
| 14 | Obesity/dh, pc, th |
| 15 | Obesity, Morbid/pc, dh, th |
| 16 | Diet Therapy/ |
| 17 | Diet, Fat-Restricted/ |
| 18 | Diet, Reducing/ |
| 19 | Dietetics/ed, mt |
| 20 | (diet or diets or dieting).ti,ab. |
| 21 | (low calorie or hypocaloric or calorie control*).ti,ab. |
| 22 | (health* adj1 eating).ti,ab. |
| 23 | (diet* adj2 (modific* or therapy or intervention* or strateg* or program* or  management or scheme*)).ti,ab. |
| 24 | (nutrition adj2 (modific* or therapy or intervention* or strateg* or program* or management or scheme*)).ti,ab. |
| 25 | (weight adj3 (modific* or therapy or intervention* or strategy* or program* or management or scheme*)).ti,ab. |
| 26 | (Success* or Los* or facilitat* or change or outcome or positive or favourable or predictor or achiev* or adhere* or compliance).ab,ti. |
| 27 | (Fail* or drop-out or dropout or barrier* or obstacle* or attrition or challeng*).ab,ti. |
| 28 | 9 or 10 |
| 29 | 11 or 12 or 13 or 14 or 15 or 16 or 17 or 18 or 19 or 20 or 21 or 22 or 23 or 24 or 25 |
| 30 | 26 or 27 |
| 31 | 8 and 29 |
| 32 | 30 and 31 |
| 33 | 28 and 32 |
| 34 | 8 and 28 and 29 and 30 |
| 35 | Adult/ |
| 36 | 34 and 35 |
| 37 | (interview: or experience:).mp. or qualitative.tw |
| 38 | 36 and 37 |
